# A predicted model-aided one-step classification–multireconstruction algorithm for X-rayfree-electron laser single-particle imaging

**DOI:** 10.1107/S2052252524007851

**Published:** 2024-08-28

**Authors:** Zhichao Jiao, Zhi Geng, Wei Ding

**Affiliations:** a Laboratory of Soft Matter Physics, Institute of Physics Chinese Academy of Sciences Beijing100190 People’s Republic of China; b Beijing Synchrotron Radiation Facility, Institute of High Energy Physics Chinese Academy of Sciences Beijing100049 People’s Republic of China; c University of Chinese Academy of Sciences Beijing 100049 People’s Republic of China; Harima Institute, Japan

**Keywords:** single-particle imaging, X-rayfree-electron lasers, classification algorithm, orientation determination algorithm

## Abstract

Ultrafast, high-intensity X-ray free-electron lasers can perform diffraction imaging of single protein molecules. Various algorithms have been developed to determine the orientation of each single-particle diffraction pattern and reconstruct the 3D diffraction intensity. Most of these algorithms rely on the premise that all diffraction patterns originate from identical protein molecules. However, in actual experiments, diffraction patterns from multiple different molecules may be collected simultaneously. Here, we propose a predicted model-aided one-step classification–multireconstruction algorithm that can handle mixed diffraction patterns from various molecules. The algorithm uses predicted structures of different protein molecules as templates to classify diffraction patterns based on correlation coefficients and determines orientations using a correlation maximization method. Tests on simulated data demonstrated high accuracy and efficiency in classification and reconstruction.

## Abstract 2

A predicted model-aided one-step classification–multireconstruction algorithm for X-ray free-electron laser single-particle imaging is proposed. The algorithm is capable of processing mixed diffraction patterns from multiple molecules, classifying diffraction patterns by different molecules, determining their orientations and reconstructing multiple 3D diffraction intensities, in one step.

## 1. Introduction

The X-ray free-electron laser (XFEL) generates ultrashort and extremely strong pulses enabling the capture of single-particle diffraction signals before radiation damage takes place (Chapman *et al.*, 2014 ▸). The first 3D reconstruction of a biomacromolecule from XFEL single-particle diffraction patterns was achieved using the Giant Mimivirus, attaining a structural resolution of 125 nm (Seibert *et al.*, 2011 ▸; Ekeberg *et al.*, 2015 ▸). After that, more and more successful 3D reconstructions of smaller molecules, such as the Melbourne virus (Lundholm *et al.*, 2018 ▸), Rice Dwarf virus (Munke *et al.*, 2016 ▸; Kurta *et al.*, 2017 ▸), bacteriophage PR772 (Reddy *et al.*, 2017 ▸; Assalauova *et al.*, 2020 ▸) and protein *Escherichia coli* GroEL (Ekeberg *et al.*, 2024 ▸) have been reported at much better resolutions. These advancements in research have experimentally validated the feasibility of single-particle imaging (SPI) techniques using XFELs. In SPI experiments, isolated particles in random orientations are injected into the X-ray pulses and the 2D diffraction patterns can be recorded in a 2D detector. The crucial step of the data processing for XFELs is finding the orientations of the recorded diffraction patterns in reciprocal space and reconstructing the 3D diffraction intensity.

Researchers have proposed many algorithms to determine the orientation of single-particle diffraction patterns. Some of the methods focus on finding the common lines or arcs along the intersection of pairs of patterns and then determine the relative orientations of all patterns (Shneerson *et al.*, 2008 ▸; Bortel & Tegze, 2011 ▸; Yefanov & Vartanyants, 2013 ▸). Manifold embedding methods try to map the diffraction patterns in high-dimensional manifest space to a 3D space of orientations (Fung *et al.*, 2008 ▸; Giannakis *et al.*, 2012 ▸). Correlation-based approaches do not find the orientation of each diffraction pattern. Instead, these methods reconstruct 3D diffraction intensity by calculating the intensity correlations of diffraction patterns (von Ardenne *et al.*, 2018 ▸; Zhao *et al.*, 2024 ▸). The multi-tiered iterative phasing method can find the orientations of patterns and recover the diffraction phases simultaneously (Donatelli *et al.*, 2017 ▸). Several methods employ the concept of expectation maximization to iteratively refine the orientations of diffraction patterns by comparing them with continuously updated 3D diffraction intensities, such as the correlation maximization (CM) algorithm (Tegze & Bortel, 2012 ▸, 2021 ▸) and the expansion maximization compression algorithm (Loh & Elser, 2009 ▸; Ayyer *et al.*, 2016 ▸). In our past work, we have introduced a predicted model-aided algorithm for orientation determination and phase retrieval, which has been successfully tested on various simulated datasets (Jiao *et al.*, 2024 ▸).

Although there are many methods that have demonstrated considerable effectiveness in dealing with XFEL data, almost all of them share a common hypothesis that all diffraction patterns are from identical particles. However, this is not always the case. In many cases, polymers or protein complexes could be formed from different kinds of monomers at room temperature and pressure, or some complexes undergo spontaneous dissociation after purification (Xu & Dang, 2022 ▸; Liu & Wang, 2023 ▸), which is a phenomenon commonly noted in cryo-electron microscopy (cryo-EM) studies. Fortunately, cryo-EM collects 2D projections of particles, allowing researchers to easily separate monomers and polymer/complexes through direct visual observation. But in single-particle diffraction experiments, the 2D diffraction patterns are noisy and non-intuitive, making it a significant challenge to figure out whether a pattern originates from a monomer or polymer/complexes. Therefore, a reconstruction algorithm that can handle mixed diffraction patterns from various molecular types is crucial for the practical application of single-molecule imaging techniques.

In this paper, we develop a predicted model-aided classification–reconstruction algorithm that can classify different molecules from mixed diffraction patterns. The predicted structures were introduced as templates to classify diffraction patterns and the CM algorithm was employed to iteratively optimize the orientations and 3D diffraction intensities. Simulated data tests demonstrate that our algorithm achieves very high accuracy in classifying mixed diffraction patterns, successfully identifying their orientations and reconstructing the 3D diffraction intensity. Moreover, our algorithm allows for the one-step 3D reconstruction of multiple 3D diffraction intensities, thereby substantially increasing computational efficiency.

## 2. Methods

### 2.1. Simulation of diffraction patterns

Based on diffraction theory, a diffraction pattern corresponds to a spherical section cut by the Ewald sphere through the 3D intensity in reciprocal space. Given the diffraction conditions, including the X-ray wavelength, the distance from the sample to the detector, and the physical shape of the detector, one can determine the reciprocal space vector **q** corresponding to each pixel in the diffraction pattern. The diffraction intensity can be calculated using the following formula:
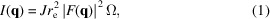
where *J* is the incident X-ray photon fluence, *r*_e_ is the classical electron radius and Ω is the solid angle subtended by the corresponding pixel on the detector. *F*(**q**) is the structure factor calculated by performing Fourier transform of the protein electron density map. Considering the non-uniform coordinates of diffraction points in reciprocal space, a non-uniform fast Fourier transform (NUFFT) (Fessler & Sutton, 2003 ▸; Geng *et al.*, 2021 ▸) was employed to avoid interpolation errors. The orientation of each diffraction pattern is entirely random through the random selection of Euler angles for molecular rotation.

Two mixtures of protein systems were used to evaluate our algorithm. One is SPARTA protein (Gao *et al.*, 2024 ▸), which is a short prokaryotic argonaute protein and the associated TIR-APAZ proteins. It forms three molecular configurations: monomers, dimers and tetramers, with respective residue counts of 1023, 2046 and 4092. The resolution of the simulated diffraction pattern is 6.6 Å. The mixed diffraction patterns include 20 000 patterns from monomers, 10 000 patterns from dimers and 5000 patterns from tetramers. The other is the binary protein complex platelet integrin αIIb–β3 (Adair *et al.*, 2023 ▸), and the mixed diffraction patterns originated from complex integrin αIIb–β3, monomers of integrin αIIb and integrin β3. The resolution of the simulated diffraction pattern is 13.1 Å. The mixed diffraction patterns include 20 000 patterns of the integrin αIIb–β3 complex, and 10 000 patterns each for the dissociated monomers, integrin αIIb and integrin β3.

Poisson noise was introduced into the diffraction patterns, and the intensity of the central 10 × 10 pixels was removed to simulate a beam stop. In the simulated light source, each pulse incorporates 2 × 10^12^ photons with a spot diameter of 0.1 µm. Considering the fluctuations in the photon flux of real light sources, a Gaussian fluctuation with a standard deviation of 10% was introduced into the photon flux. A more detailed description of the simulation diffraction parameters is provided in Table 1 ▸. The simulated diffraction patterns are shown in Figs. S1–S2 of the supporting information.

### 2.2. Obtaining initial templates using *AlphaFold*2

**Figure 1:**
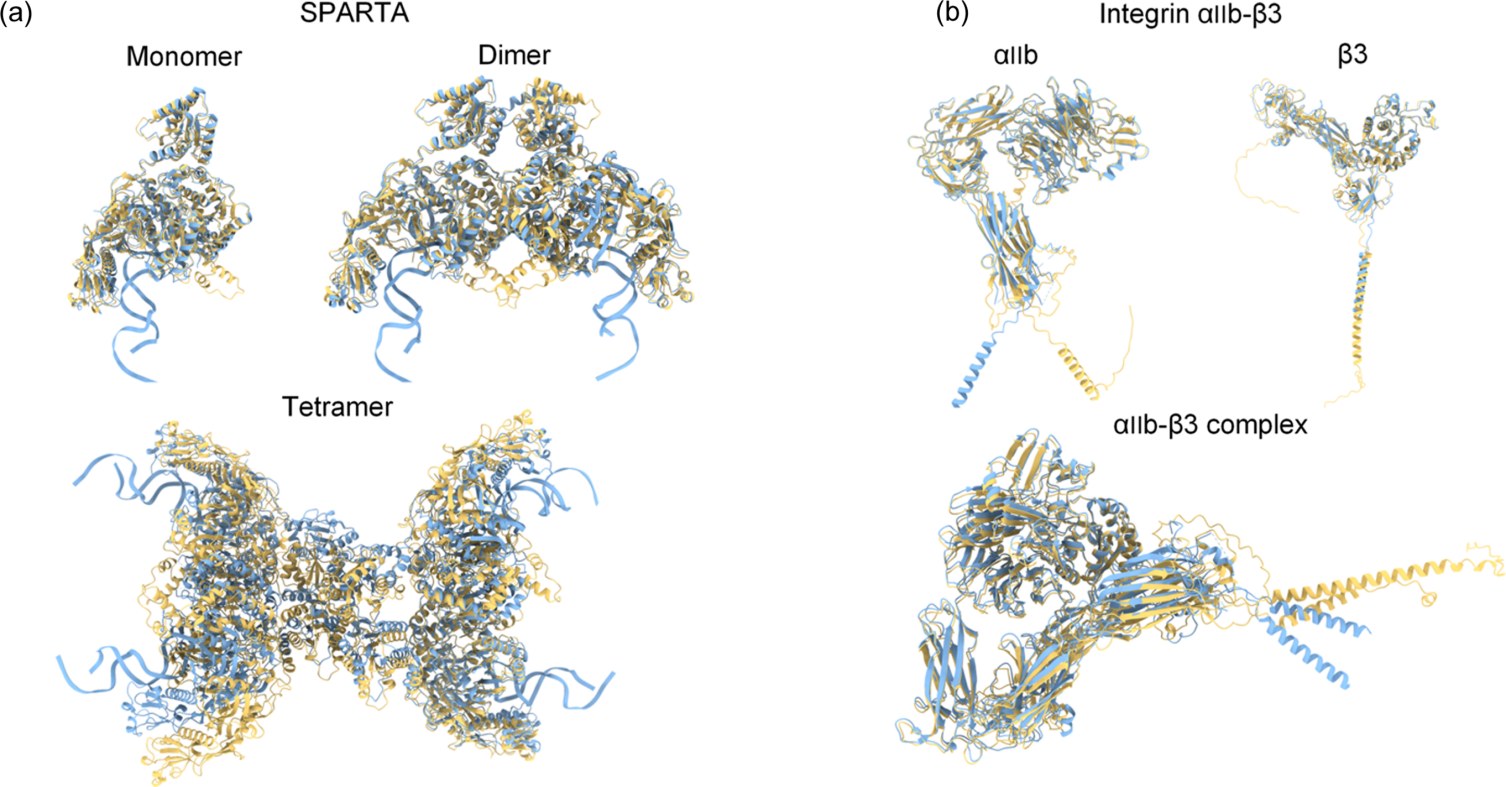
Comparison of predicted and real molecular structures. Blue – real structure; yellow – predicted structure. (*a*) SPARTA protein system: monomers, dimers and tetramers. (*b*) Integrin αIIb–β3 system: the integrin αIIb–β3 complex and its dissociated monomers, integrin αIIb and integrin β3.

The structures of SPARTA monomer, dimer, tetramer and the integrin αIIb–β3 complex were predicted by local *AlphaFold*2 (Jumper *et al.*, 2021 ▸). And the predicted structures of integrin αIIb and integrin β3 were directly downloaded from the *AlphaFold*2 database (https://alphafold.com/). A comparison between the predicted and experimental structures is illustrated in Fig. 1 ▸, where yellow represents the predicted structures and blue denotes the experimental structures. The figure demonstrates that, for the SPARTA system, the predicted structure of the monomer is the most accurate, well matching the experimental structure. As the protein size increases, the prediction becomes more challenging, with the predicted structure of the tetramer exhibiting significant deviation from the experimental structure. Another notable distinction in the SPARTA system is that the experimental structure includes the gRNA–tDNA fragment, which is absent in the predicted structure. The root-mean-square deviations (RMSDs) between the predicted and actual structures was calculated using *Phenix* (Adams *et al.*, 2010 ▸), and the results for the monomer, dimer and tetramer are 2.18, 3.81 and 10.68 Å, respectively. In the integrin αIIb–β3 system, a flexible α-helix present in both monomers leads to the primary differences between the predicted and experimental structures. The RMSD for the integrin αIIb–β3 complex, integrin αIIb monomer and integrin β3 monomer are 3.05, 3.17 and 1.58 Å, respectively.

After obtaining the predicted structures, each atom was treated as a Gaussian peak to generate an electron density map. By sampling in reciprocal space based on the parameters used in the simulated diffraction, the electron density map is transformed into reciprocal space via NUFFT, and then squared to calculate the 3D diffraction intensity. These 3D diffraction intensities in reciprocal space are to be utilized as initial templates in the one-step classification–multireconstruction algorithm.

### 2.3. One-step classification-multireconstruction algorithm

**Figure 2:**
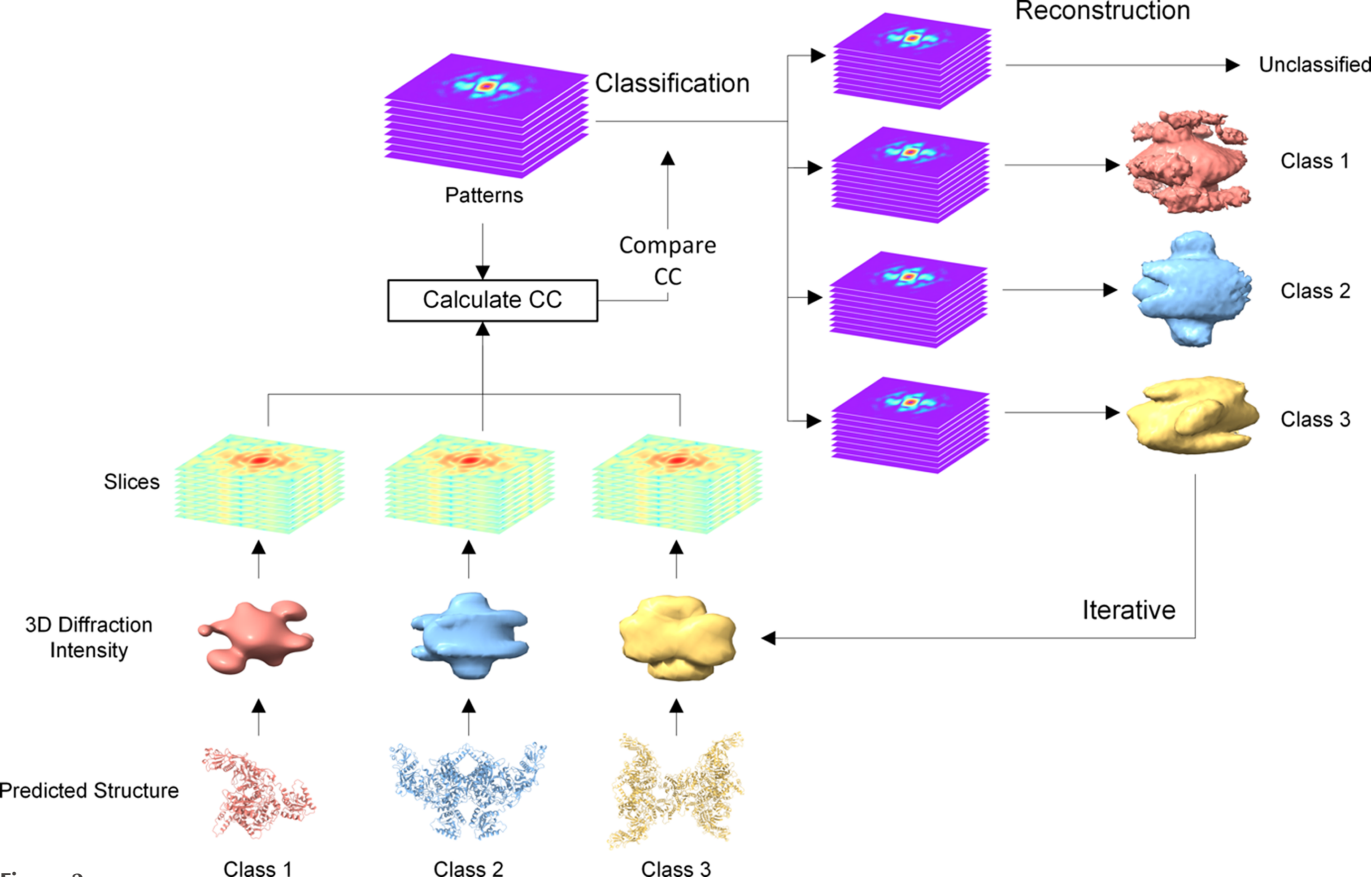
Diagram of the one-step classification–multireconstruction algorithm. This process classifies mixed diffraction patterns by comparing them with multiple templates from predicted structures while simultaneously determining the orientation to reconstruct multiple 3D diffraction intensities. Classification results and orientations are continuously refined through iteration.

Our algorithm utilizes predicted 3D diffraction intensities as the initial templates for classification according to the similarity between each diffraction pattern and the templates. At the same time, the best orientation for each diffraction pattern is determined, and then diffraction patterns within the same class are merged based on their best orientation into a set of updated 3D intensities, serving as templates for the next round of classification. After several iterations, diffraction patterns from different molecules will be classified and reconstructed at once. Fig. 2 ▸ provides a concise overview of the algorithm’s core procedure. Specifically, our algorithm is divided into the following steps:

(1) Preprocessing diffraction patterns and 3D diffraction intensities. All diffraction images and predicted 3D intensities are downsampled by a factor of two to enhance the signal-to-noise ratio and improve computational efficiency.

(2) Cutting the 3D diffraction intensities in all possible orientations. Orientations are determined using Euler angles, and by appropriately selecting the values of Euler angles, uniform sampling of all possible orientations within the 3D space can be ensured. Details of the 3D orientation sampling are provided in Appendix *A*[App appa]. Based on the parameters used in the simulated diffraction, the Ewald sphere is calculated, rotated to the specified orientation and the 3D diffraction intensities are then cut to yield the 2D diffraction slices.

(3) Calculating the correlation coefficient between each diffraction pattern and all the 2D diffraction slices. The Pearson correlation coefficient (Lee Rodgers & Nicewander, 1988 ▸) is employed to evaluate the similarity between a diffraction pattern and a slice. The formula for the coefficient is provided as follows:
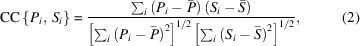
where
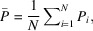
where *P_i_* and *S_i_* represent the corresponding pixels in the pattern and the slice, respectively. For each diffraction pattern, the correlation coefficient is calculated with all slices derived from each 3D intensity. CC_max_(*n*, *m*) represents the maximum value among the correlation coefficients between the *n*th diffraction pattern and all slices of the *m*th 3D intensity. To accelerate computation, both the diffraction pattern and the slice have been transformed into polar coordinates and then FFT is employed; details are provided in Appendix *A*[App appa].

(4) Classification of diffraction patterns according to their correlation coefficients. For each diffraction pattern, we have calculated a set of CC_max_, where the number of CC_max_ is equal to the number of classes. For each diffraction pattern, its associated CC_max_ is sorted, where 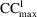 denotes the highest value within that pattern, 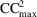 represents the second highest within the same pattern and so on. Only diffraction patterns whose CC_max_ satisfy the following criteria will be classified:
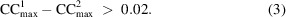
This diffraction pattern will be assigned to the class corresponding to 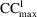 and used to reconstruct 3D diffraction intensity. Any diffraction pattern failing to satisfy equation (3) will be assigned as unclassified and will not be used for reconstructing any 3D intensities. Setting this threshold for the correlation coefficient can effectively lower the proportion of incorrectly classified diffraction patterns. During the early iterations, it is common for many diffraction patterns to have comparable CC_max_ values across various classes, hence being assigned as unclassified. However, with ongoing iterations, the quality of 3D intensities is enhanced, diminishing the number of unclassified diffraction images.

(5) Reconstruction of updated 3D intensities. Following classification, diffraction patterns within each class are utilized to reconstruct a new series of 3D intensities, based on their best orientation indicated by the CC_max_. In the beginning iterations, the number of diffraction patterns used for reconstruction is relatively low due to a high number of unclassified diffraction patterns. As iterations proceed, the number of diffraction patterns in each class will gradually increase, and the best orientation of each diffraction pattern will become increasingly accurate, thereby improving the quality of the reconstructed 3D intensities.

(6) Iterate from step (2) to (5) until the classification and best orientation of each diffraction pattern stabilize. Of note, at every classification step, not only the unclassified diffraction patterns but all diffraction patterns undergo reclassification. Throughout this process, some misclassified diffraction patterns will be corrected.

### 2.4. Computational environment

The algorithm was written in C, Python and Bash, utilizing MPI parallelization to accelerate performance. The computations were executed on a computer featuring an Intel Core i7-12700 processor, which has 12 cores and 20 threads. All calculations were performed on the CPU, without using any GPU resources. For a single iteration of the algorithm on 35 000 diffraction patterns and 3 classes, the runtime was approximately 18 min. Molecular graphics were made using *UCSF Chimera* (Pettersen *et al.*, 2021 ▸).

## 3. Results and discussion

### 3.1. Mixed diffraction patterns of monomers, dimers and tetramers

To assess the efficacy of the algorithm, we first chose the SPARTA protein system. Mixed diffraction patterns were simulated, with 20 000 originating from monomers, 10 000 from dimers and 5000 from tetramers. The parameters used for the simulation are presented in Table 1 ▸. Predicted diffraction intensities from three types of protein molecules are employed as initial templates for classifying mixed diffraction patterns.

**Figure 3:**
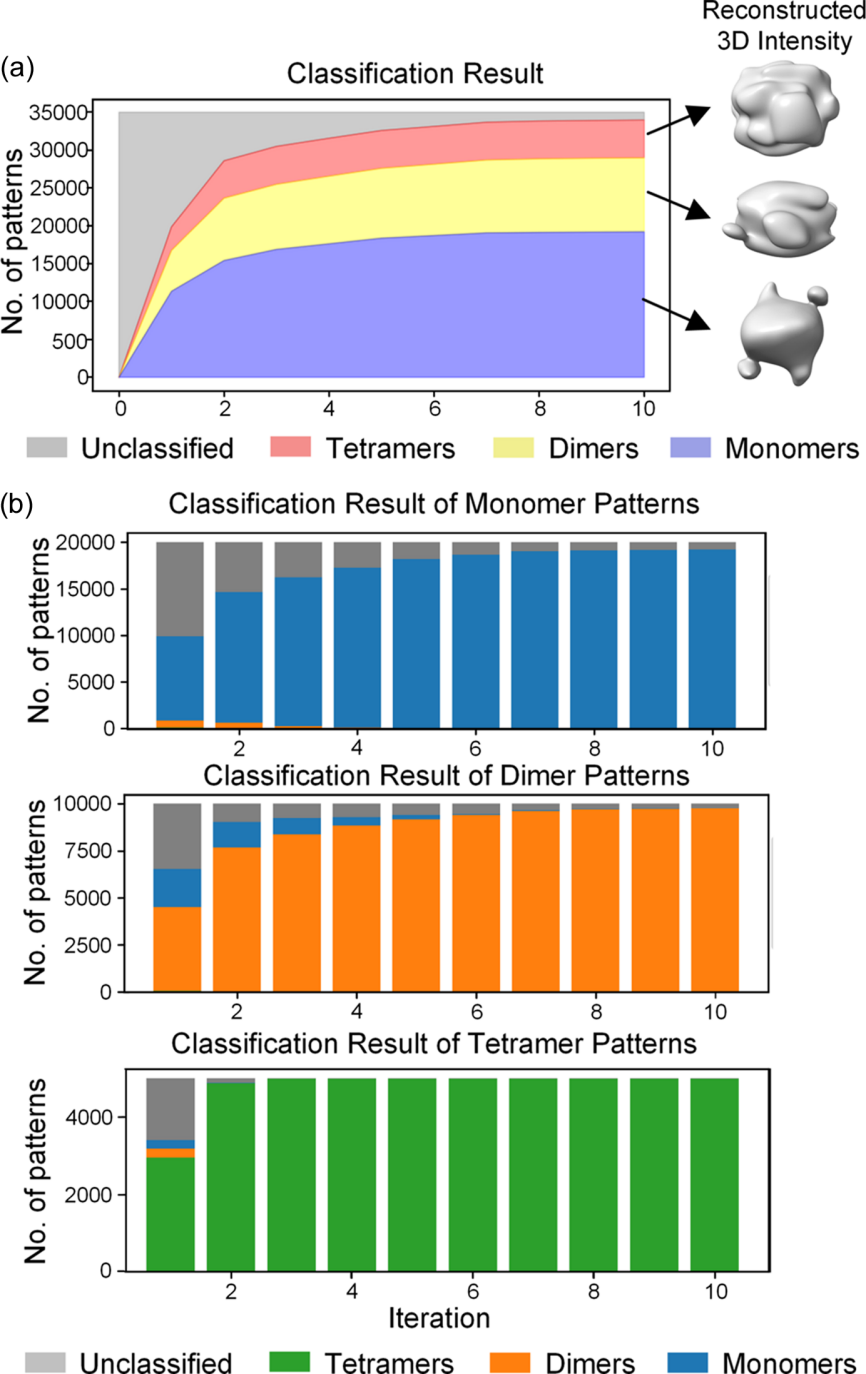
Classification results of the SPARTA system. (*a*) Classification results of mixed diffraction patterns during the iterative process. Red – diffraction patterns classified as tetramers; yellow – diffraction patterns classified as dimers; blue – diffraction patterns classified as monomers; gray – unclassified diffraction patterns. (*b*) Classification results of different molecular diffraction patterns during the iterative process. The first row contains 20 000 patterns diffracted by monomers, the second row contains 10 000 patterns diffracted by dimers and the third row contains 5000 patterns diffracted by tetramers. Green – diffraction patterns classified as tetramers; orange – diffraction patterns classified as dimers; blue – diffraction patterns classified as monomers; gray – unclassified diffraction patterns.

Following the initial classification, 11 355 diffraction patterns were identified as monomers, 5395 as dimers and 3105 as tetramers, as depicted in the Fig. 3 ▸(*a*). Among all successfully classified diffraction patterns, the accuracy of classification reached a relatively high 83.00%, as shown in the Table 2 ▸. The trade-off is that a significant proportion, specifically 15 145 diffraction patterns (43% of all patterns), were assigned as unclassified after the first round of classification. Simultaneously with classification, the optimal orientation was identified for each diffraction pattern. Based on the classification and orientation results, three new 3D intensities were reconstructed to be used as templates in the next iteration.

In the second iteration, a greater number of diffraction patterns were successfully classified: 15 418 as monomers, 8232 as dimers and 4938 as tetramers. Moreover, the classification accuracy increased to 93.07%, indicating that the 3D intensities reconstructed in the first round were superior to the predicted intensities. In subsequent iterations, simultaneous advancements were made in the number of successfully classified diffraction patterns, classification accuracy, orientation precision and the quality of reconstructed 3D intensities. After ten iterations, 33 970 diffraction patterns (97% of all patterns) were successfully classified, with a remarkably high accuracy of 99.94%.

Tracking the classification results of diffraction patterns from a single type of molecule throughout the iterative process is highly insightful. As illustrated in Fig. 3 ▸(*b*), each row represents all diffraction patterns from the same molecule, with different colors indicating classification into distinct classes. In the first classification, a significant portion of diffraction patterns from three type of molecule remained unclassified, with a minor fraction classified to an incorrect class. Among them, the dimer diffraction patterns showed the highest misclassification rate, with 20.46% of the patterns misidentified as monomers. This could be attributed to the similarity in diffraction intensities between dimers and monomers at certain orientations. As the iterations progressed, misclassified diffraction patterns quickly disappeared. After ten iterations, a small portion of diffraction patterns from monomers and dimers remained unclassified. On the other hand, among the 5000 diffraction patterns of tetramers, 4999 were correctly classified as tetramers. This can be explained by the larger molecular size of tetramers, resulting in higher signal-to-noise ratios in their diffraction patterns, making them easier to classify.

**Figure 4:**
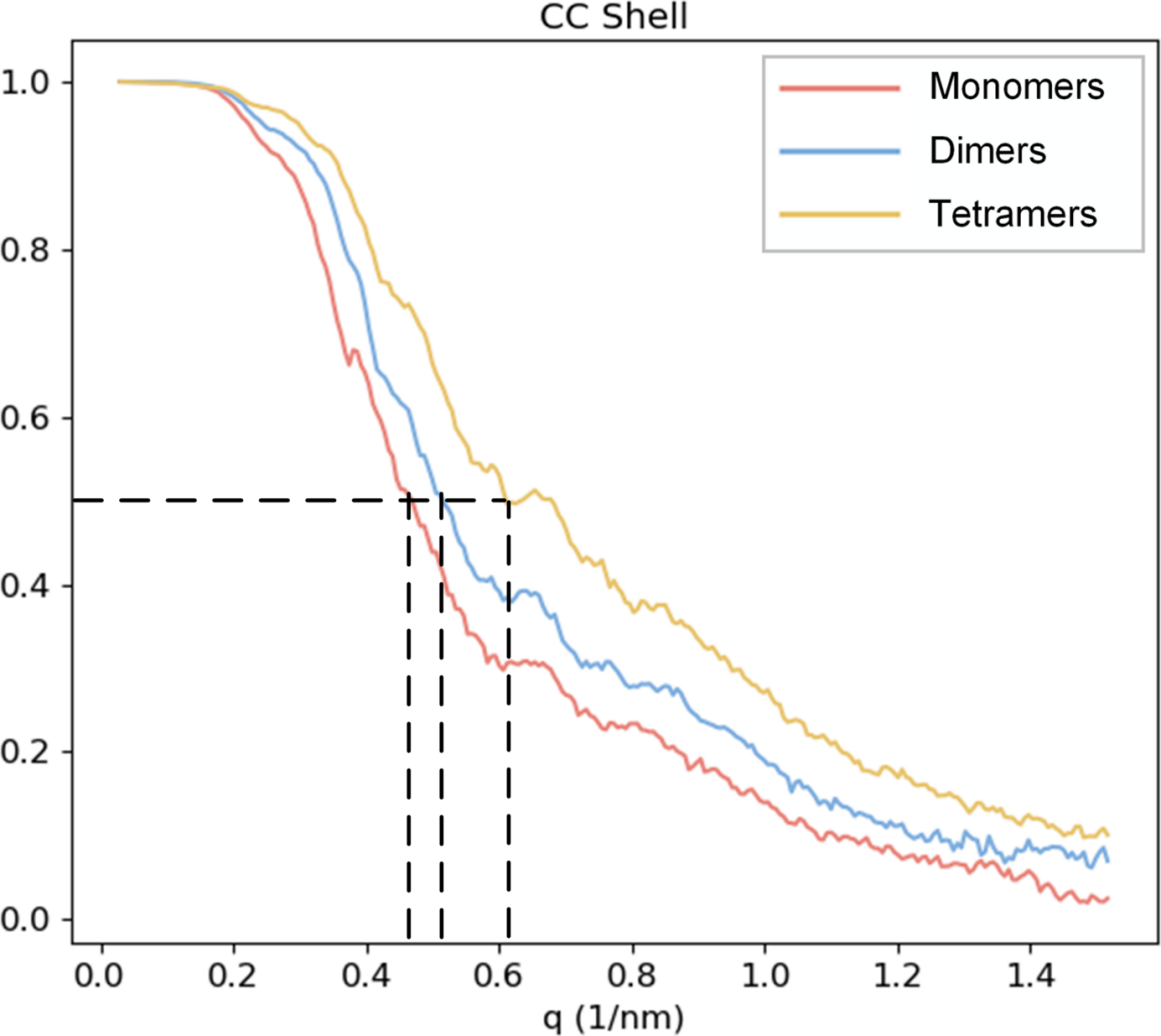
Correlation coefficients between reconstructed diffraction intensities and real intensities of the SPARTA protein system in different resolution shells. Red curve – monomer; blue – dimer; yellow – tetramer. Dotted line indicates the resolution of the reconstructed 3D diffraction intensity, where the CC drops to 0.5. The resolution of the monomer, dimer and tetramers are 21, 19 and 16 Å, respectively.

We calculated the correlation coefficients between the reconstructed 3D diffraction intensities from the final results and the true intensities across various resolution shells, as shown in Fig. 4 ▸. Despite having the fewest diffraction patterns, the tetramers achieved the highest final resolution of 16 Å due to their stronger diffraction signals. The resolutions of the 3D intensities for dimers and monomers are 19 and 21 Å, respectively.

### 3.2. Mixed diffraction patterns of complex and dissociated monomers

The algorithm was also tested using the integrin αIIb–β3 complex system for further evaluation. We simulated the dissociation of complexes, including 20 000 diffraction patterns of the integrin αIIb–β3 complex, and 10 000 diffraction patterns each for the dissociated monomers, integrin αIIb and integrin β3. The parameters used for the simulation are presented in Table 1 ▸.

**Figure 5:**
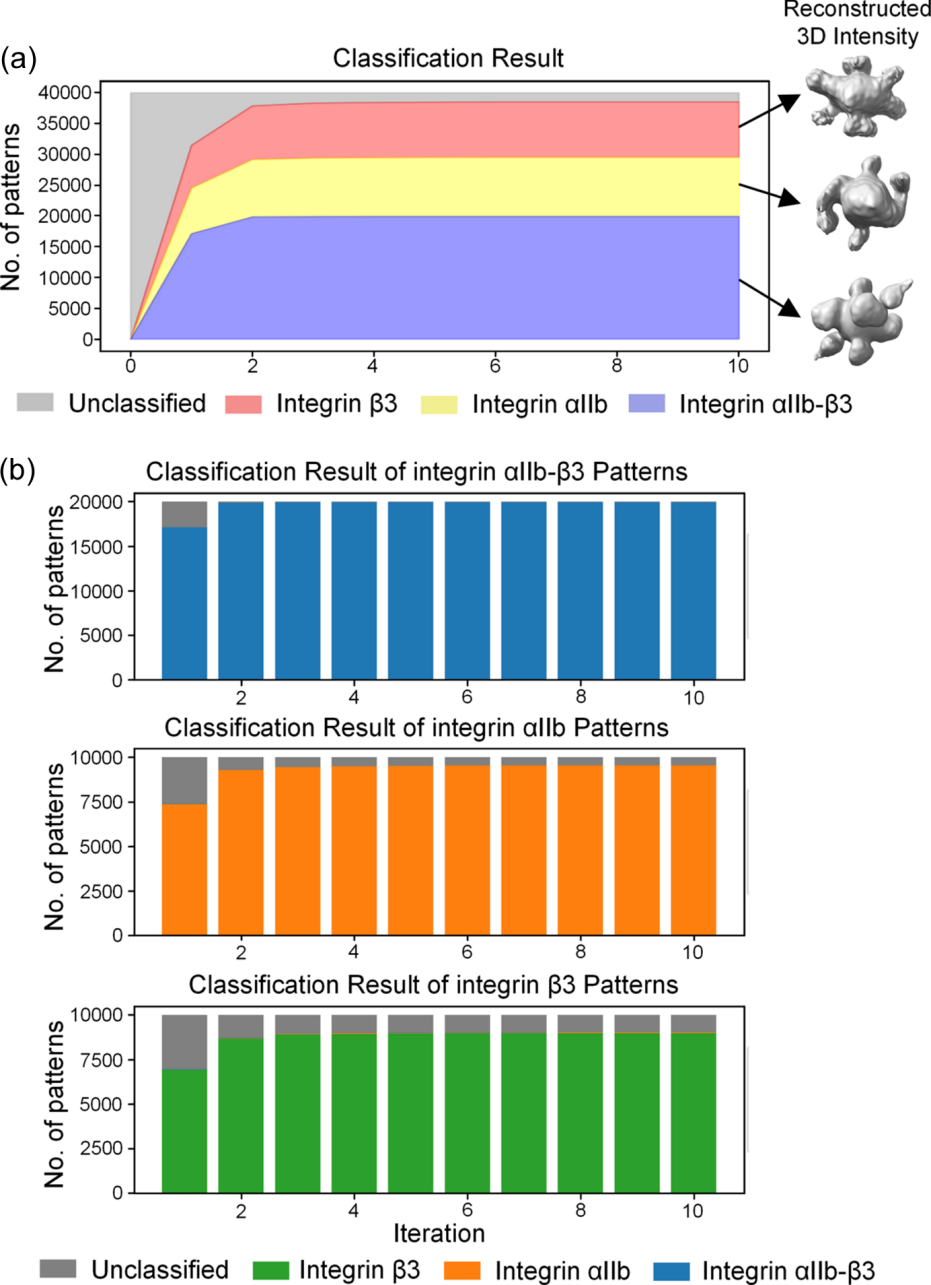
Classification results of the integrin αIIb–β3 system. (*a*) Classification results of mixed diffraction patterns during the iterative process. Red – diffraction patterns classified as the integrin β3 monomer; yellow – diffraction patterns classified as the integrin αIIb monomer; blue – diffraction patterns classified as the integrin αIIb–β3 complex; gray – unclassified diffraction patterns. (*b*) Classification results of different molecular diffraction patterns during the iterative process. The first row contains 20 000 patterns diffracted by the integrin αIIb–β3 complex, the second row contains 10 000 patterns diffracted by the integrin αIIb monomer and the third row contains 10 000 patterns diffracted by the integrin β3 monomer. Green – diffraction patterns classified as the integrin β3 monomer; orange – diffraction patterns classified as the integrin αIIb monomer; blue – diffraction patterns classified as the integrin αIIb-β3 complex; gray – unclassified diffraction patterns.

In the first iteration, 17 117 diffraction patterns were classified as complex, while 7406 and 6950 diffraction patterns were classified as monomer αIIb and β3, respectively, as shown in Fig. 5 ▸(*a*). And the number of unclassified diffraction patterns was 8527 (21% of all patterns). Among all successfully classified diffraction patterns, the accuracy rate of classification reached as high as 99.71%, as shown in Table 3 ▸. The excellent outcomes of the initial classification are potentially due to the more accurate predicted structures of these three proteins, which provided improved 3D intensity templates for classification. As the iterations progressed, an increasing number of diffraction patterns were successfully classified, with corresponding enhancements in classification accuracy. By the second iteration, 37 867 diffraction patterns had been successfully classified, achieving an accuracy rate of 99.80%. The algorithm was nearing convergence, with only minor changes in classification results in subsequent iterations.

The classification results for diffraction patterns of each type of molecule are shown in Fig. 5 ▸(*b*) separately. The diffraction intensities of the whole complexes are the highest, hence the classification of their patterns is relatively simple, with 19 915 of 20 000 diffraction patterns accurately classified as complexes after ten iterations. And the diffraction patterns of monomers αIIb and β3 exhibit weaker signals, posing a greater challenge for classification. The remaining unclassified diffraction patterns are predominantly from these two types of molecules. Despite the challenges, the percentage of unclassified particles remains below 5%.

**Figure 6:**
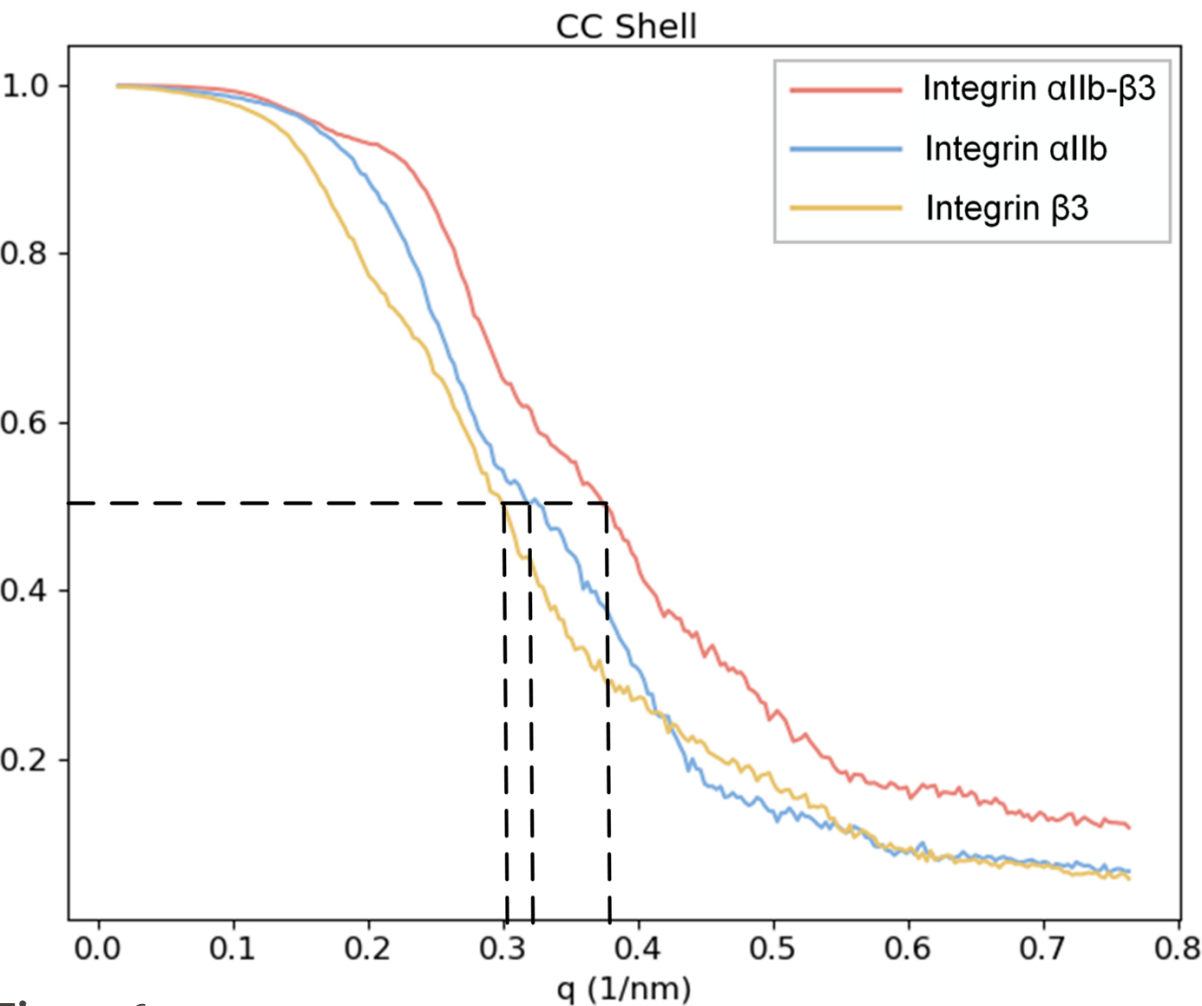
Correlation coefficients between reconstructed diffraction intensities and real intensities of the integrin αIIb–β3 system in different resolution shells. Red curve – integrin αIIb–β3 complex; blue – integrin αIIb monomer; yellow – integrin β3 monomer. Dotted line indicates the resolution of the reconstructed 3D diffraction intensity, where CC drops to 0.5. The resolution of the integrin αIIb–β3 complex, integrin αIIb monomer and integrin β3 monomer are 27, 31 and 33 Å, respectively.

The orientation of each diffraction pattern was determined simultaneously with classification, leading to the reconstruction of three 3D diffraction intensities based on these orientations. Fig. 6 ▸ displays the correlation coefficients between the reconstructed 3D intensities and the true 3D intensities across different resolution shells. The complexes have the highest number of diffraction patterns and strongest diffraction signals, resulting in the highest resolution of the reconstructed 3D intensities, reaching 27 Å. For the two monomers, the residue count of integrin αIIb is slightly higher than that of integrin β3 (with values of 1008 and 762, respectively), and the quantity of diffraction patterns utilized in the reconstruction is also slightly greater for integrin αIIb (9576 compared with 9006), resulting in a resolution of 31 Å, marginally higher than the 33 Å resolution of integrin β3.

## 4. Conclusions

This research presented a one-step classification–multireconstruction algorithm designed to separate different molecules from mixed diffraction patterns while simultaneously reconstructing multiple 3D diffraction intensities. The classification is achieved by comparing correlation coefficients between a diffraction pattern and various templates generated from predicted structures. At the same time, the orientation of each diffraction pattern is determined by the correlation coefficient and used to update the 3D intensity template.

We set a threshold for the difference in correlation coefficients, marking diffraction patterns with approximate similarity to several templates as unclassified. This strategy effectively minimizes the quantity of unclassified diffraction patterns, thereby avoiding potential cascading errors and enhancing the stability and robustness of the algorithm. In this paper, a threshold of 0.02 was used, selected based on experience and recommended as a suitable value for most cases. However, this threshold can be adjusted in different cases, depending on the trade-off between the number of patterns used in the reconstruction and the accuracy of the classification. Moreover, testing indicated that the probability of misclassifying diffraction patterns is greatest in the first iteration and decreases with further iterations. Therefore, an automatic method for selecting and adjusting the threshold is beneficial. For example, using a larger threshold at the start of the iterations ensures classification accuracy, and then reducing the threshold during the iterations allows more diffraction patterns to be classified.

The effectiveness and accuracy of this algorithm in classification and orientation determination were validated using simulated data. Unfortunately, the absence of experimental data prevents us from testing our algorithm with real data at this time. Recently, Ekeberg *et al.* (2024 ▸) utilized XFELs to capture diffraction patterns of single protein molecules and reconstructed their 3D structures. This significant advancement propelled SPI from viral to protein specimens, marking a major leap forward. With the ongoing development and improvement of XFEL sources and single-particle experimental techniques, it is certain that they will be applied to a broader range of protein samples. We look forward to applying our algorithm to real experimental data in the future, aiding in the single-particle reconstruction of proteins.

## Supplementary Material

Supporting figures. DOI: 10.1107/S2052252524007851/it5036sup1.pdf

## Figures and Tables

**Figure 7:**
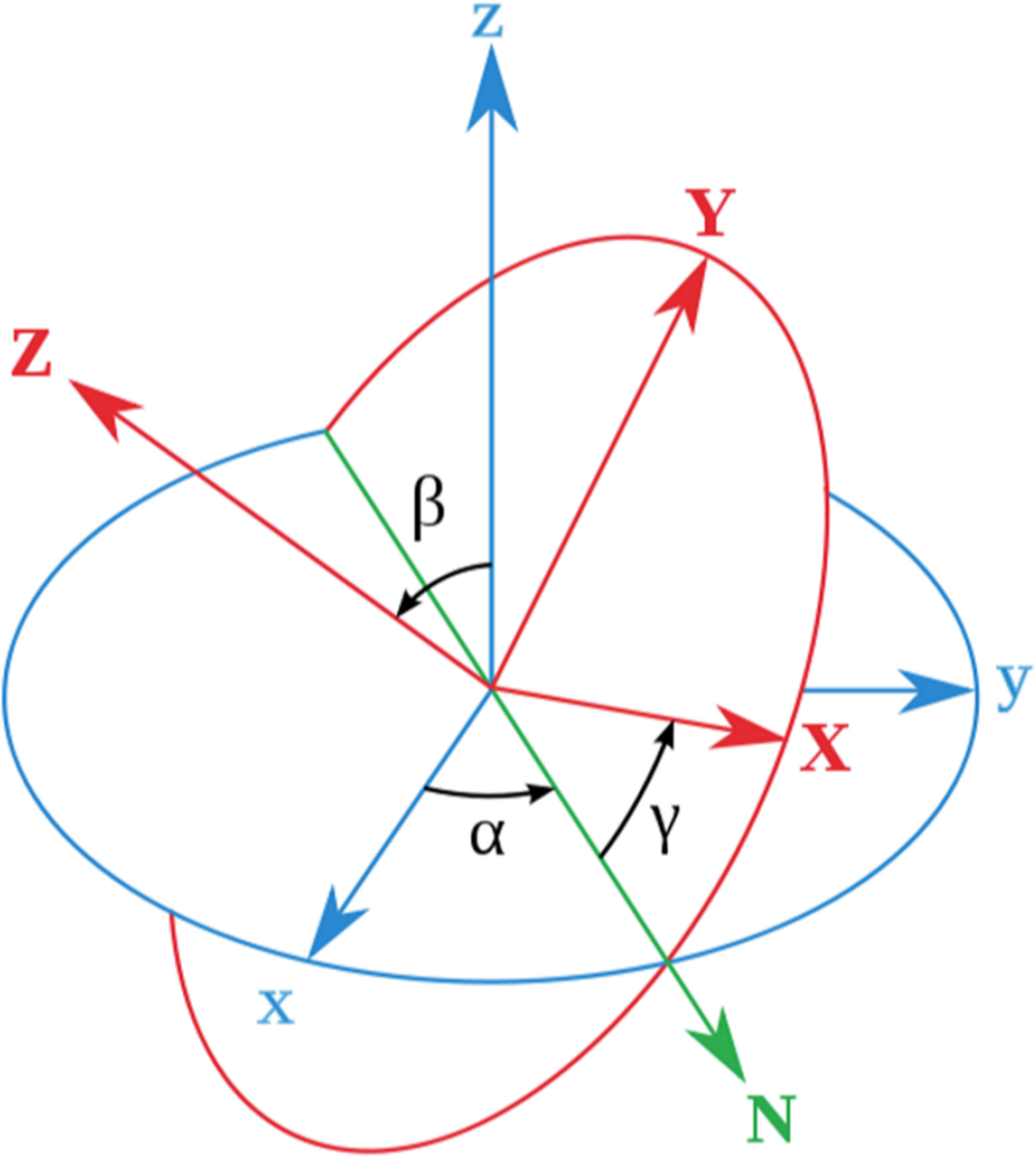
Diagram of Euler angles used in orientation sampling. The red *Z* axis represents the direction of the incident X-rays. A specified set of α and β defines a diffraction pattern, and γ determines the self-rotation angle.

**Table 1:** Parameters used in the simulation of diffraction patterns

	SPARTA system (monomer, dimer, tetramer)			Integrin αIIbβ3 system (αIIb–β3, αIIb, β3)		
Protein	SPARTA monomer	SPARTA dimer	SPARTA tetramer	Integrin αIIb–β3	Integrin αIIb	Integrin β3
PDB code	8isz †	8k9g †	8it1 †	8t2v ‡	8t2v (chain A)	8t2v (chain B)
No. of amino acid residues	1023	2046	4092	1770	1008	762
No. of patterns	20000	10000	5000	20000	10000	10000
XFEL wavelength (Å)	1			1		
Photon flux (photons per pulse)	2 × 10^12^			2 × 10^12^		
Beam focus size (µm)	0.1			0.1		
Detector size (pixels)	512 × 512			512 × 512		
Pixel size (µm)	300			300		
Beam stop size (pixels)	10 × 10			10 × 10		
Sample-to-detector distance (m)	0.5			1		
Resolution of pattern (Å)	6.6			13.1		

† From the work by Gao *et al.* (2024 ▸).

‡ From the work by Adair *et al.* (2023 ▸).

**Table 2:** Classification accuracy of the SPARTA system Classification accuracy = number of correct classified patterns/number of successful classified patterns.

	1	2	3	4	5	6	7	8	9	10
Classification accuracy (%)	83.00	93.07	96.40	98.24	99.17	99.78	99.87	99.93	99.94	99.94

**Table 3:** Classification accuracy of the integrin αIIb–β3 complex system Classification accuracy = number of correct classified patterns/number of successful classified patterns.

	1	2	3	4	5	6	7	8	9	10
Classification accuracy (%)	99.71	99.80	99.81	99.82	99.82	99.81	99.81	99.81	99.81	99.81

## Data Availability

Our code is open-source. All code used in this article can be found at https://github.com/ZhichaoJiao/SPI_class_multireconstruct.git. The authors confirm that the data supporting the findings of this study are available within the article and its supplementary materials.

## Appendix A Orientation sampling and correlation coefficient calculation

The orientation in 3D space can be represented by a set of Euler angles α, β, and γ, as illustrated in Fig. 7 ▸. We define the incident X-ray direction as the red *Z*-axis direction. Consequently, molecular rotation about the red *Z* axis does not modify the diffraction pattern, but merely induces self-rotation. Therefore, γ is treated differently from α and β; a specified set of α and β defines a diffraction pattern, and γ determines the self-rotation angle. For a complete exploration of every orientation within 3D space, the ranges of α, β and γ are set to 2π, π and 2π, respectively. To ensure uniform sampling, it is necessary to specifically design the sampling intervals of α and β, as described by the following equation:

where Δα and Δβ represent the sampling intervals of α and β, respectively. In our test, Δβ is set to 0.1 radians, corresponding to 1273 distinct orientations throughout the entire 3D space.

For each diffraction pattern, the correlation coefficient must be calculated with all slices at every self-rotation angle. Diffraction patterns and slices in Cartesian coordinates are converted into polar coordinates to reduce the amount of computation. Subsequently, angular normalization within each radius determined is applied to diffraction patterns and slices in polar coordinates, resulting in a mean of zero and a variance of one. Accordingly, the Pearson correlation coefficient for a diffraction pattern *P_n_*(*r*, θ) and a slice *S_R_*(*r*, θ) at a specified self-rotation angle γ is represented by

where *P_n_*(*r*, θ) represents the *n*th pattern in polar coordinates, *S_R_*(*r*, θ + γ) represents a slice with an *R* orientation, which has self-rotated by an angle of γ. Specifically, due to the absence of low-resolution data and the poor signal-to-noise ratio at high resolution in the diffraction patterns, both regions are excluded from correlation coefficient calculations by setting boundaries with *r*_min_ and *r*_max_. According to the cross-correlation theorem, by performing Fourier transforms on both the diffraction patterns and slices, the correlation coefficients for all self-rotation angles can be computed at once:

where **θ** is a vector of azimuthal angles with a range of 2π, the operator  represents 1D Fourier transforms about vector **θ**,  represents the conjugate of 1D Fourier transforms and  is the inverse 1D Fourier transforms. CC(*n*, *R*) contains a set of correlation coefficients, each corresponding to a different self-rotation angle, with the highest one chosen to represent the correlation between the diffraction pattern and the slice.
