# Supplementary material for: A predicted model-aided one-step classification–multireconstruction algorithm for X-rayfree-electron laser single-particle imaging
Source: IUCrJ. 2024 Aug 28;11(Pt 5):891–900. doi: 10.1107/S2052252524007851 (PMC11364030; doi:10.1107/S2052252524007851)
Supplement: Supplementary file 1 [file m-11-00891-sup1.pdf]

IUCrJ

Volume 11 (2024)

Supporting information for article:

A predicted model-aided one-step classification-multireconstruction algorithm for X-ray free-electron laser single-particle imaging

Zhichao Jiao, Zhi Geng and Wei Ding

Simulated diffraction patterns of different molecules

Although theoretically the detector is sufficiently large to collect high-resolution diffraction signals, the majority of pixels in the high-resolution region receive zero photons due to insufficient diffraction intensity. Photons are primarily concentrated in the low-resolution region.

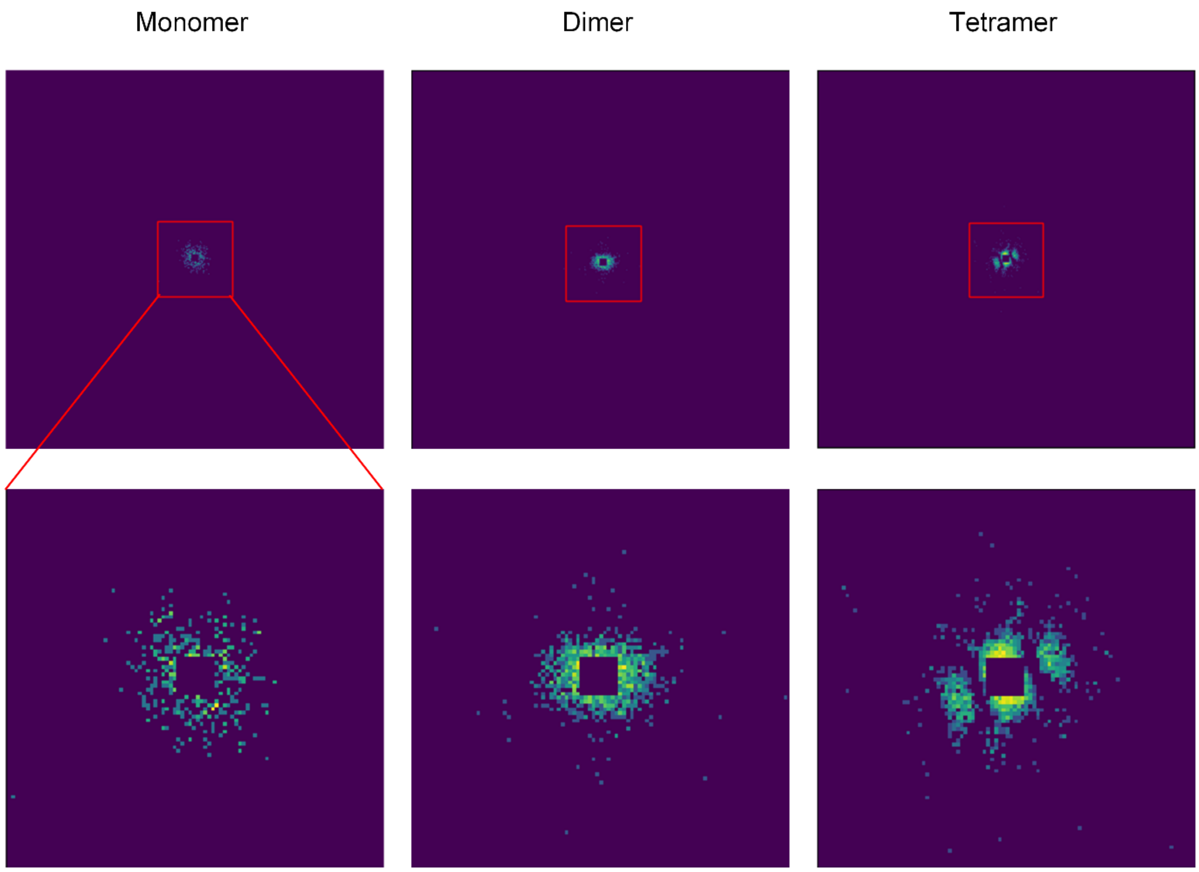

Figure S1 Diffraction patterns of SPARTA monomer, dimer and tetramer molecules. Note that the resolution at the edge of the diffraction pattern is 6.6 Å.

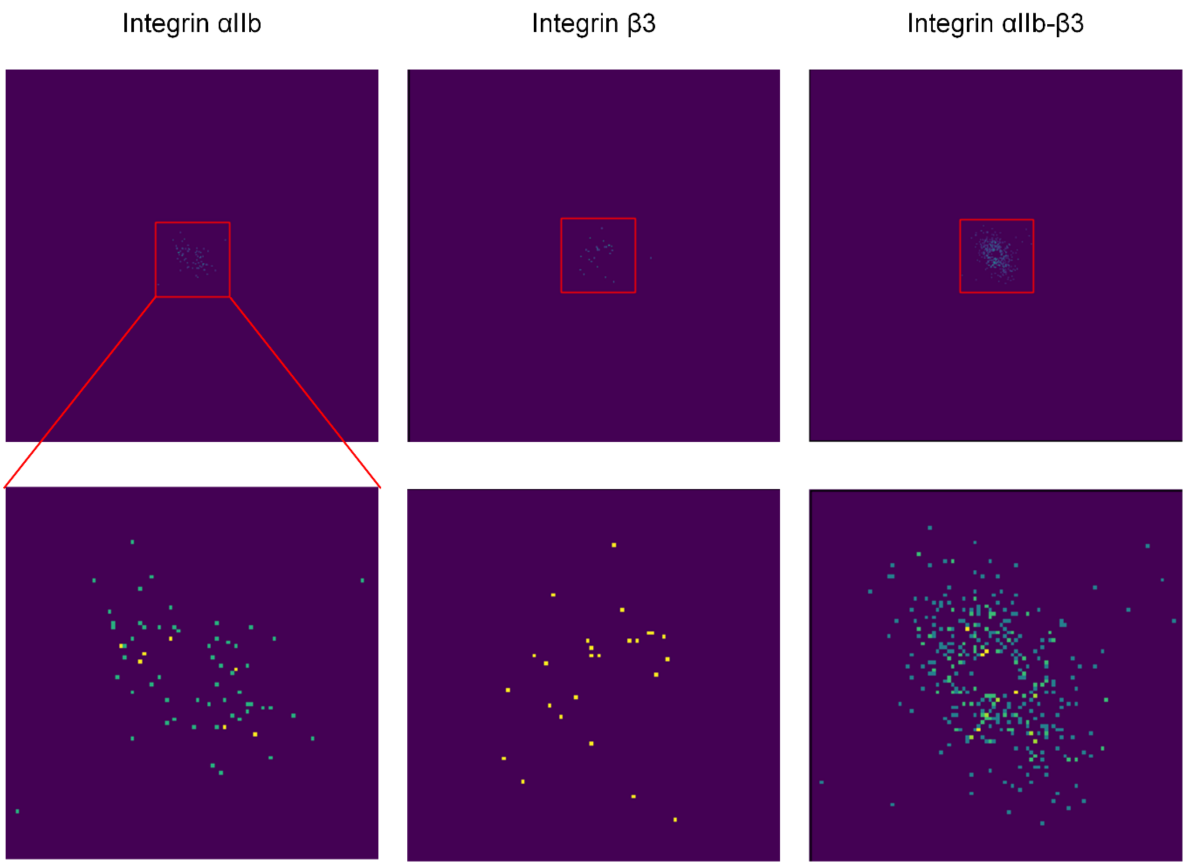

Figure S2 Diffraction patterns of integrin αIIb, integrin β3 and integrin αIIb-β3 molecules. Note that the resolution at the edge of the diffraction pattern is 13.1 Å.

The variation of CC_{mean} throughout the iterative process

CC_{mean} is the mean value of CC_{max}^{1} for all diffraction patterns at each iteration in the CM algorithm (see equation (3)), is used to monitor the convergence of the orientation determination algorithm.

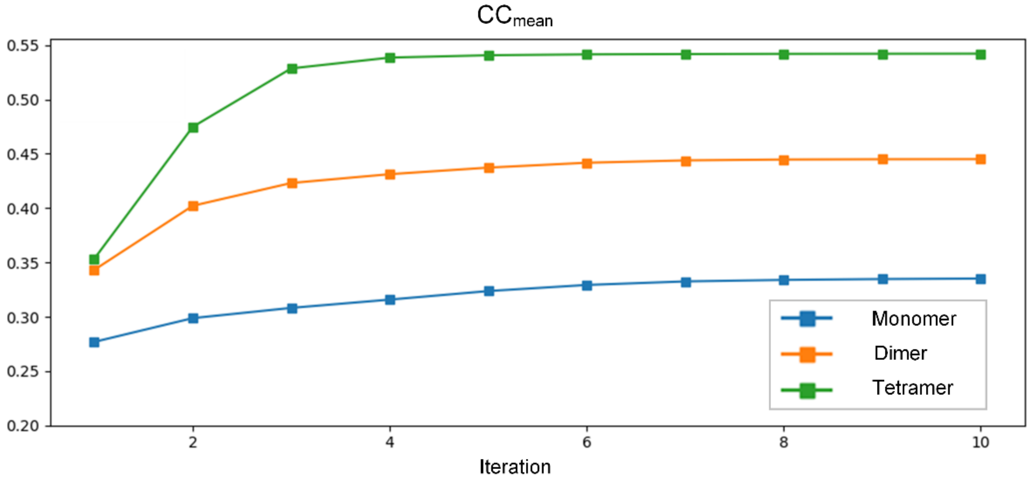

Figure S3 Changes in the CC_{mean} of SPARTA monomers, dimers, and tetramers. Note that before the 10th iteration, the CC_{mean} no longer increases, indicating that the algorithm has converged.

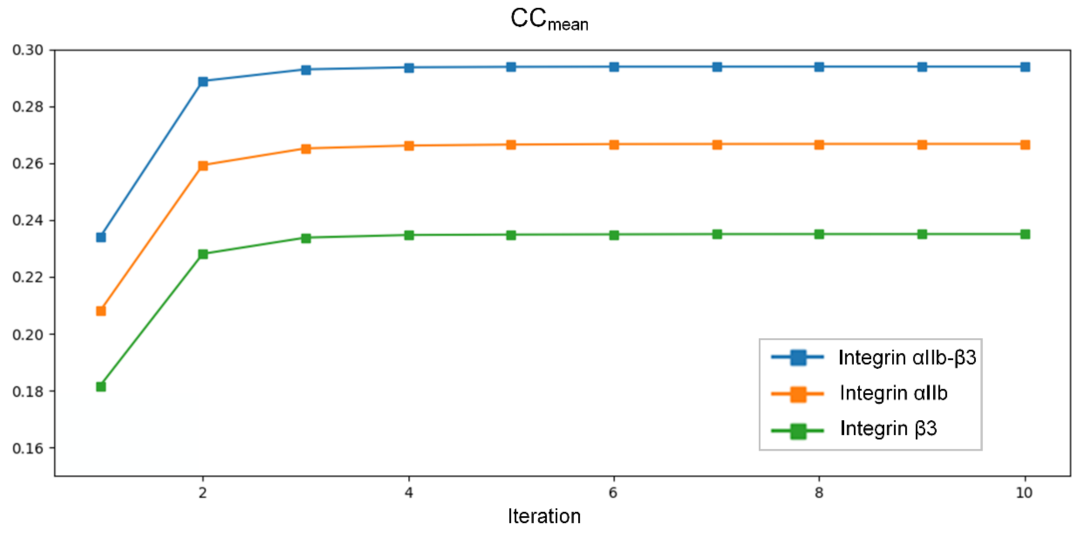

Figure S4 Changes in the CC_{mean} of integrin αIIb, integrin β3 and integrin αIIb-β3. Note that before the 10th iteration, the CC_{mean} no longer increases, indicating that the algorithm has converged.
